# Anion Binding Based on Hg_3_ Anticrowns as Multidentate Lewis Acidic Hosts

**DOI:** 10.1021/acs.inorgchem.2c00921

**Published:** 2022-08-01

**Authors:** Oliver Loveday, Jesús Jover, Jorge Echeverría

**Affiliations:** † Secció de Química Inorgànica, Departament de Química Inorgànica i Orgànica and Institut de Química Teòrica i Computacional (IQTC-UB), Universitat de Barcelona, Martí i Franquès 1-11, 08028 Barcelona, Spain; ‡ Departamento de Química Inorgánica, Instituto de Síntesis Química y Catálisis Homogénea (ISQCH), CSIC-Universidad de Zaragoza, Pedro Cerbuna 12, 50009 Zaragoza, Spain

## Abstract

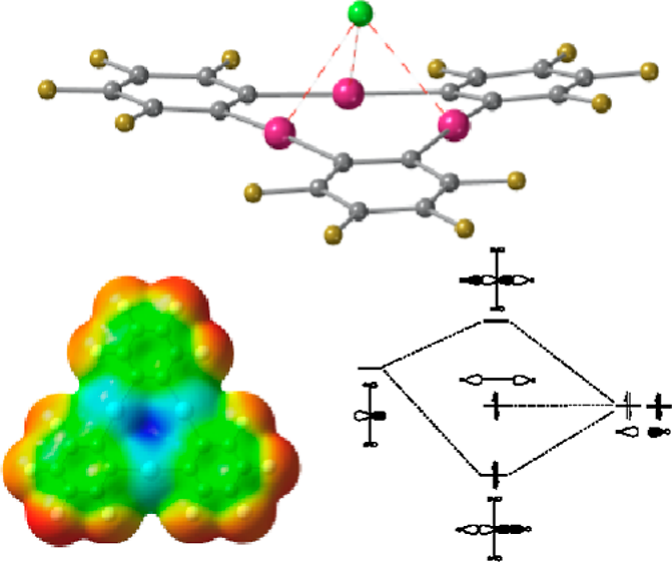

We present herein a combined structural and computational analysis of the anion binding capabilities of perfluorinated polymercuramacrocycles. The Cambridge Structural Database (CSD) has been explored to find the coordination preference of these cyclic systems toward specific Lewis bases, both anionic and neutral. Interaction energies with different electron-rich species have been computed and further decomposed into chemically meaningful terms by means of energy decomposition analysis. Furthermore, we have investigated, by means of the natural resonance theory and natural bond orbital analyses how the orbitals involved in the interaction are key in determining the final geometry of the adduct. Finally, a generalization of the findings in terms of the molecular orbital theory has allowed us to understand the formation of the pseudo-octahedral second coordination sphere in linear Hg(II) complexes.

## Abstract 2

Hg_3_ anticrowns are capable of binding a wide range of Lewis bases, both anionic and neutral, via their three metal centers, where not only electrostatics but also the orbitals involved in the interaction determine the final geometry and stability of the adducts. Moreover, the molecular orbital theory allows the rationalization of the formation of a pseudo-octahedral second coordination sphere in d^10^ closed-shell linear complexes.

## Introduction

The design and synthesis of chemical systems that efficiently recognize and capture anions have attracted increasing attention during the last decades.^1^ Anion binding is based on the establishment of noncovalent interactions, for example, hydrogen and halogen bonds, between the anion and the molecular host.^2^ Several families of compounds have been used to capture different types of anionic systems, for instance, cryptands,^3^ cucurbiturils,^4,5^ or calixarenes.^6,7^ Recently, transition metal-based anion receptors have been investigated as promising anion capturers.^8^ These systems bind anions via their ligands or directly through the metal center by means of metal···anion interactions. Among the latter, polymetallic macrocycles make use of simultaneous interactions with multiple metal centers to improve the anion binding capacity. Recently, the capture of halide anions by cyclic (Py-M)_3_ (M = Cu, Ag, and Au) systems has been computationally studied.^9^

**Scheme 1:**
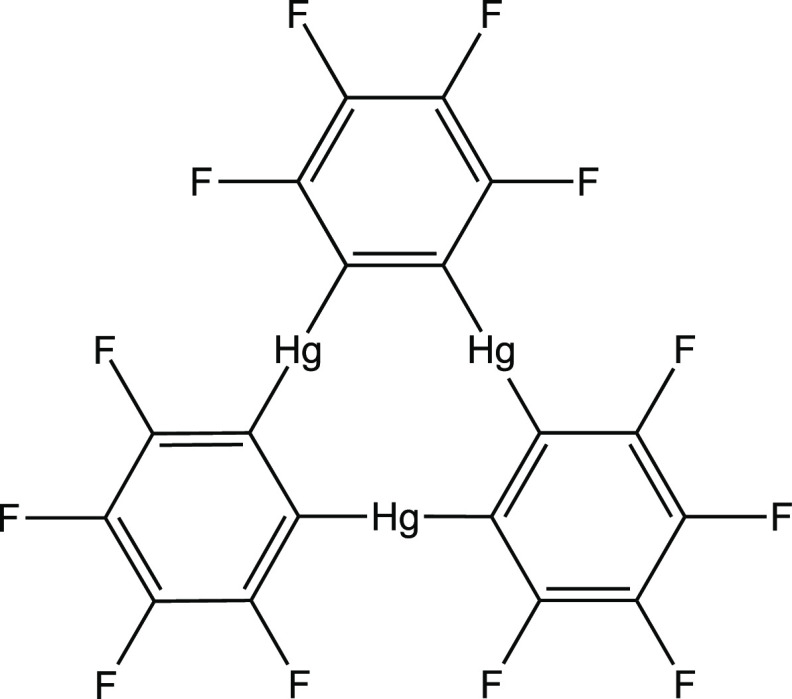
Chemical Structure of Perfluoro-*o*-phenylenemercury [(*o*-C_6_F_4_Hg)_3_]

Perfluorinated polymercuramacrocycles are a family of compounds with enhanced electrophilicity due to the presence of several Lewis acid centers that can act coordinatively to bind a wide range of anions. Moreover, the presence of the peripheric fluorine atom increases the electron density depletion in the central region of the molecule. Polymercuramacrocycles have been generally termed anticrowns since they can be seen as the electron-deficient equivalents of crown ethers.^10^ Here, we narrow our focus on perfluoro-*o*-phenylenemercury [(*o*-C_6_F_4_Hg)_3_; see Scheme 1]. Ever since it was first synthesized in the 1960s,^11^ the anion binding capabilities of this compound have been extensively investigated.^12^ The high degree of preorganization along with the simultaneous action of three Lewis acidic centers makes (*o*-C_6_F_4_Hg)_3_ a versatile and efficient anion capturer.

Herein, we intend to study, by means of computational tools, the interaction of this anticrown not only with several anionic species but also with neutral electron-rich species that are often used as solvents. We will analyze the nature of the interaction with different Lewis bases (LBs) and how electrostatics and, in particular, orbital interactions determine the final geometry and stability of the adduct.

## Results and Discussion

### Experimental Crystal Structures

**Figure 1:**
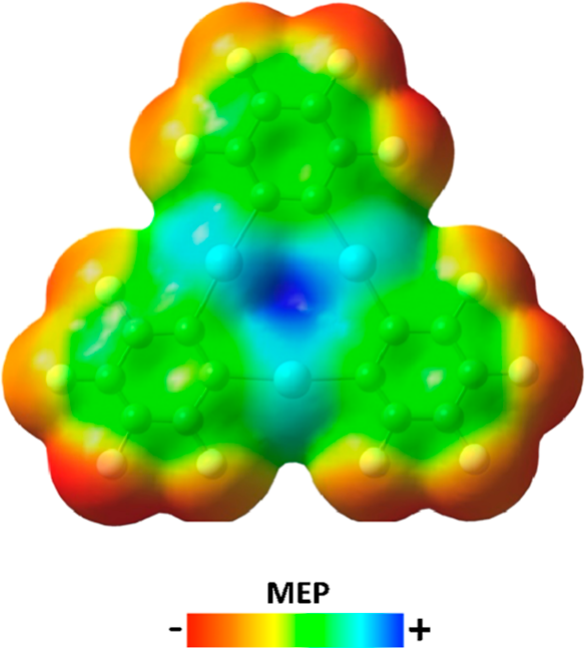
MEP map on the *s* = 0.001 isosurface of (*o*-C_6_F_4_Hg)_3_.

**Figure 2:**
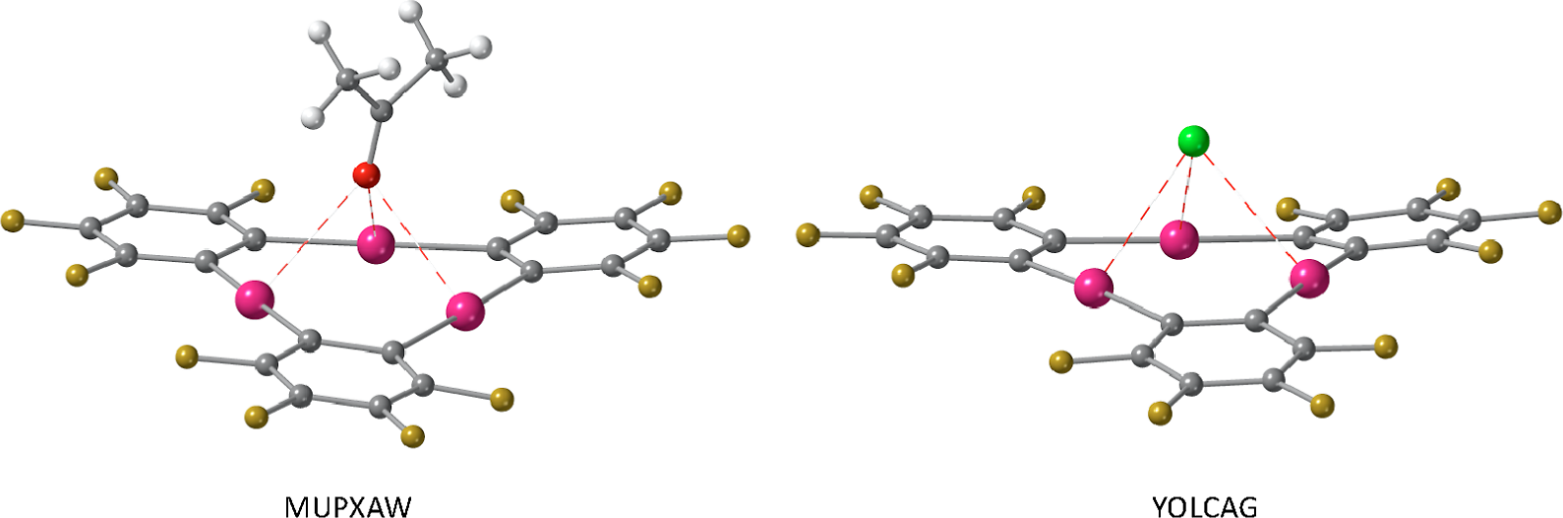
Short Hg···O and Hg···Cl contacts (red dashed lines) found in the crystal structures of {[(*o*-C_6_F_4_Hg)_3_](Me–CO–Me)_3_} (MUPXAW)^27^ and [(CH_2_)_10_(NH_2_)_2_(NH)_2_]{[(*o*-C_6_F_4_Hg)_3_]Cl_2_} (YOLCAG),^28^ respectively.

The molecular electrostatic potential (MEP) of the (*o*-C_6_F_4_Hg)_3_ molecule clearly displays a region of electron density depletion (*V*_s,max_ = +60.3 kcal/mol) located at the center of the triangle formed by the three Hg atoms (Figure 1). This electrophilic region is significantly exposed due to the planarity of the molecule and, thus, is able to be reached by electron-rich species, both anionic and neutral. In fact, there are many examples in the Cambridge Crystallographic Database (CSD) in which a LB interacts with the electron-depleted region involving three Hg···LB distances shorter than the sum of the van der Waals radii.^13^ Here, the (*o*-C_6_F_4_Hg)_3_ molecule behaves as a three-dentate Lewis acid via its three Hg(II) centers. On the other hand, we have observed a marked variability in the nature of the interacting LB, including both neutral and anionic species such as benzene (ABELUO),^14^ naphthalene (MOXMIV),^15^ triphenylene (MOXMOB),^15^ corannulene (IXOBAZ),^16^*closo*-dodecaborate (ACEREF),^17^ dimethyl sulfoxide (CAMFOM),^18^ methyl parathion (CEJNUB),^19^ ferrocene (FANNUE),^20^ and nickelocene (FANPAM)^20^ via their Cp ligands, nitrate (HIMHUI)^21^ and nitrobenzene via the O atoms (REWZID),^22^ and dimethylsulfide (UKUQEW),^23^ carbon disulfide (PAGLIT),^24^ coordinated pentaphosphole (JOVHOT)^25^ and pentarsolyl (JOVVOH),^25^ and polyynes through their triple bonds (JEGLEN).^26^ In Figure 2, two selected examples are shown in which the LBs are dimethylketone and chloride (MUPXAW^27^ and YOLCAG,^28^ respectively).

**Figure 3:**
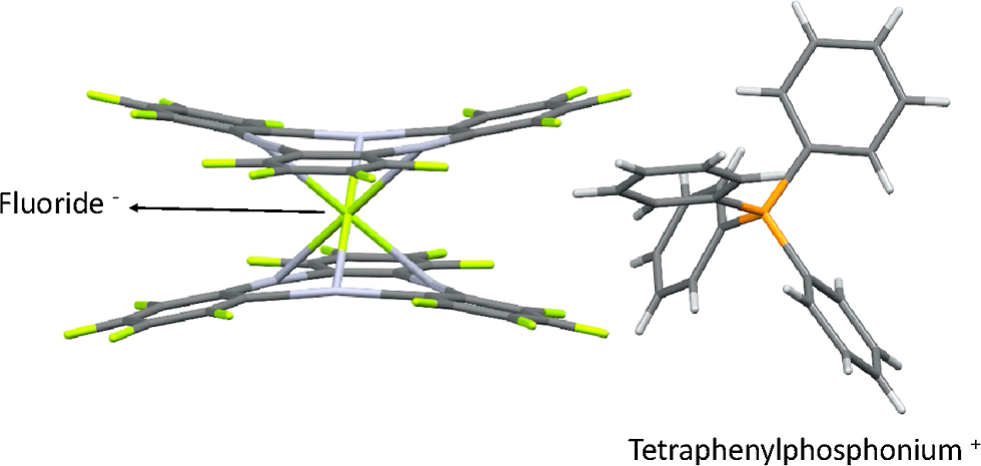
Crystal structure of [PPh_4_]{[(*o*-C_6_F_4_Hg)_3_]_2_F} (UTOZEK).^29^

It is worth noting that the halide anions in crystalline phases tend to bind Hg centers of the Hg_3_ anticrown instead of the corresponding counter-cations. For instance, in the crystal structure of [PPh_4_]{[(*o*-C_6_F_4_Hg)_3_]_2_F}, the fluoride anion is placed between two Hg_3_ anticrowns, forming a sandwich-like structure, while the large tetraphenylphosphonium interacts with the ensemble through C–H/π and C–H···F–C interactions (Figure 3).

### Geometry of the Adducts and Interaction Energies

We have performed a computational study of the interaction geometries and strength of several models composed of (*o*-C_6_F_4_Hg)_3_ and different LBs, both charged and neutral. The main results, which are compared to the experimental structures when available, are presented in Table 1. In general, the density functional theory (DFT) results fairly reproduce the geometries observed in crystal structures. For monoatomic anions, DLPNO-CCSD(T) interaction energies are considerably high and depend on the size of the anion, being maximum for hydride (−96.9 kcal/mol) and minimum for iodide (−48.8 kcal/mol). It is interesting to note that the interaction energies calculated for anions show a nice linear correlation (*R*^2^ = 0.97) with the degree of penetration between X and Hg atoms^30^ (see Figure S1 in the Supporting Information). On the other hand, for neutral LBs, the strength of the interaction diminishes significantly, ranging from −9.7 kcal/mol for water to −16.0 kcal/mol for acetone. These results are a good indicator of the capability of Hg_3_ anticrowns to be exploited as anion capturers. If we look at the equilibrium geometries, there are also some differences between anionic LBs and some of the neutral LBs. While the three Hg···LB distances for anions, acetone, and acetonitrile are practically the same, in the case of dimethyl ether, one of the computed Hg···O contacts is significantly shorter than the other two (2.949 and 3.438/3.4507 Å, respectively), which could be attributed to the establishment of CH/π interactions between a methyl group and one of the aromatic rings of the anticrown. For water, however, there are two practically identical distances (3.067 Å) and a significantly longer one (3.403 Å). Since such a geometry cannot be related with any secondary noncovalent interaction between the Hg_3_ anticrown and the LB, it must be due to some particular feature of the water–Hg interaction.

**Table 1:** Distances between Mercury Atoms and the Donor Atom of the LB and Interaction Energies Calculated after Full Geometry Optimization (B3LYP-D3BJ/def2-TZVPD) of the Adducts Formed by (*o*-C_6_F_4_Hg)_3_ and the Different LBs^a^

LB	LB···Hg exp. (Å)	refcode	LB···Hg calc. (Å)	Δ*E*_int_ (kcal/mol)
H^–^			2.126	–96.9
			2.126	
			2.126	
F^–^	2.620	UTOZEK^29^	2.470	–80.8
	2.614		2.471	
	2.647		2.471	
Cl^–^	2.994	YOLCAG^28^	2.944	–58.2
	3.005		2.944	
	3.023		2.943	
Br^–^			3.098	–53.8
			3.098	
			3.098	
I^–^	3.261	UTOMAT^29^	3.298	–48.8
	3.321		3.298	
	3.389		3.298	
H_2_O	2.821	OYAPUA^31^	3.067	–9.7
	2.928		3.067	
	3.136		3.403	
Me–O–Me			2.949	–13.4
			3.438	
			3.457	
Me–CO–Me	2.815	MUPXAW^27^	2.974	–14.4
	2.858		3.021	
	2.906		3.027	
Me–CN	2.930	WOSFAL^32^	3.113	–16.0
	2.932		3.113	
	2.989		3.114	

a Experimental distance values are given for comparison when available. Interaction energies (Δ*E*_int_) are obtained at the DLPNO-CCSD(T)/def2-TZVPD level on the DFT-optimized geometries.

**Figure 4:**
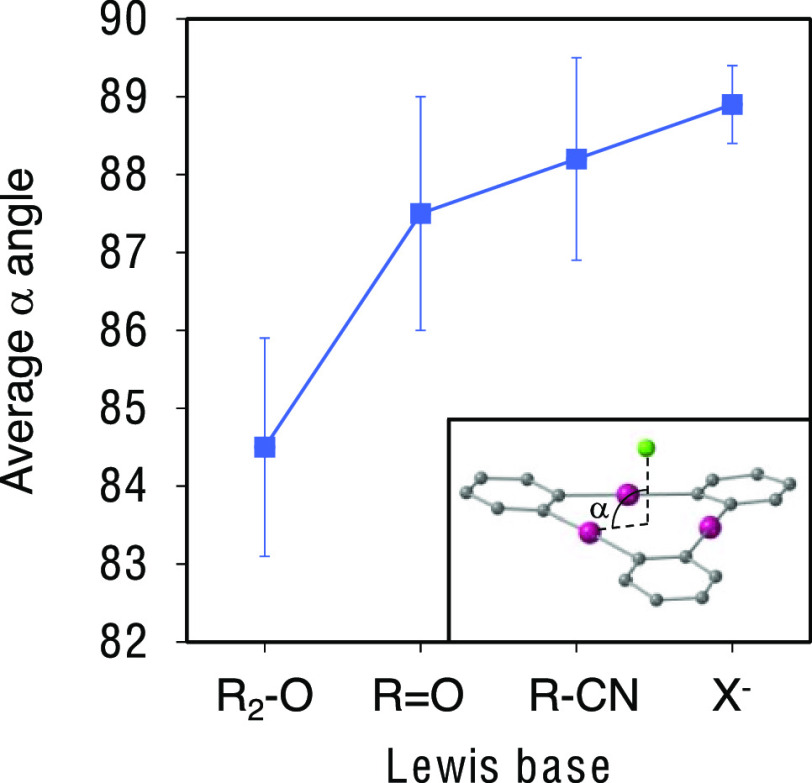
Variation of the averaged smallest Hg–centroid–LB (α) angle as a function of the nature of the donor atom.

Although this behavior is also observed in the crystal structure of [(*o*-C_6_F_4_Hg)_3_] dihydrate (OYAPUA),^31^ we wanted to be sure that the out-of-the-center displacement from our calculations is not just the consequence of a flat potential energy surface (PES) in the central region of the Hg_3_ system. To do so, several starting geometries were used in the optimizations, leading in all cases to the same equilibrium geometry, which was confirmed to be a real minimum of the PES by computation of the corresponding vibrational frequencies. Furthermore, we defined an angle α between the donor atom of the LB, the centroid of the Hg_3_ system, and one of the Hg atoms (Figure 4 inset), and we searched the CSD for short LB···Hg_3_ contacts for different families of LBs. The results representing the average values of the smallest angle α are summarized in Figure 4. It can be seen that while for halides, the angle α is very close to 90°, it diminishes (i.e., moves off the Hg_3_ center) for nitriles and ketones, the decrease being more pronounced when the donor is an ether-like sp^3^ oxygen atom (averaged smallest angle α = 84.5°). This confirms the different behaviors, both experimental and computational, of water and ethers with respect to the other studied LBs.

### Energy Decomposition Analysis

To gain further insights into the nature of the interactions, we have carried out an energy decomposition analysis (EDA) of the model systems in Table 1. We applied the ALMO-EDA scheme that decomposes the interaction energy into terms that are chemically meaningful (Δ*E*_INT_ = Δ*E*_FRZ_ + Δ*E*_POL_ + Δ*E*_CT_). The Δ*E*_FRZ_ term represents the energy changes associated with bringing together two fragments that were infinitely separated without any relaxation of their molecular orbitals and is composed of Pauli repulsion and Coulomb interactions and dispersion (Δ*E*_Pauli_, Δ*E*_Elect_, and Δ*E*_Disp_). On the other hand, Δ*E*_POL_ and Δ*E*_CT_ terms account for the energy stabilization due to intrafragment and interfragment relaxation of the MOs, respectively. Representative EDA results are shown in Table 2.

**Table 2:** EDA Results Calculated at the B3LYP-D3BJ/def2-TZVPD Level for the Adducts Formed by (*o*-C_6_F_4_Hg)_3_ and Different LBs^a^

LB	Δ*E*_Pauli_	Δ*E*_Elect_	Δ*E*_Disp_	Δ*E*_POL_	Δ*E*_CT_	Δ*E*_INT_
H^–^	296.6	–274.1	–12.9	–64.5	–50.8	–105.7
F^–^	130.9	–137.9	–4.3	–51.3	–18.1	–80.8
Cl^–^	107.1	–101.9	–12.33	–31.9	–21.8	–60.8
Br^–^	103.7	–96.5	–13.5	–28.8	–21.9	–56.9
I^–^	103.0	–91.3	–15.8	–25.2	–23.8	–53.2
H_2_O	12.7	–11.6	–5.6	–2.1	–2.0	–8.6
Me–O–Me	17.6	–12.9	–11.6	–2.8	–2.9	–12.5
Me–CO–Me	21.5	–17.5	–11.0	–4.3	–3.2	–14.5
Me–CN	17.6	–15.1	–8.1	–3.6	–3.0	–12.2

a All energies in kilocalories per mole.

It can be seen that Δ*E*_FRZ_ is in general negative, which means that electrostatics and dispersion attractive forces are able to overcome Pauli exchange repulsion. The only exception is the hydride adduct, in which the repulsive frozen energy is counterbalanced by very large orbital-based terms, namely, Δ*E*_POL_ and Δ*E*_CT_. Remarkably, both polarization and charge transfer are very important for anions. Polarization is particularly relevant for halides, representing 63.5, 52.5, 50.6, and 47.5% of all attractive terms for fluoride, chloride, bromide, and iodide, respectively. For neutral LBs, the sum of polarization and charge transfer accounts for ∼50% of all negative energy terms. These EDA results further suggest that orbital interactions, even in the case of anionic donors, play an important role in the formation of adducts.

### Molecular Orbital Picture

It seems clear now that the trend in the equilibrium geometries observed both computationally and experimentally cannot be explained by using MEP maps as the only predictors. Previous reports have also shown the incapability of MEP maps to rationalize interactions in which repulsive Coulombic forces (predicted by MEPs) are overcome by dispersion and/or orbital charge transfer (both invisible for MEPs).^33−36^ In light of this and according to our EDA results above, we wondered if the particular behavior of water toward Hg_3_ anticrowns can be the result of specific orbital interactions. In order to unveil any charge transfer process between the Lewis acidic and basic species, we first need to establish the mercury-centered molecular orbitals that are able to delocalize electron density from the LB lone pairs.

**Figure 5:**
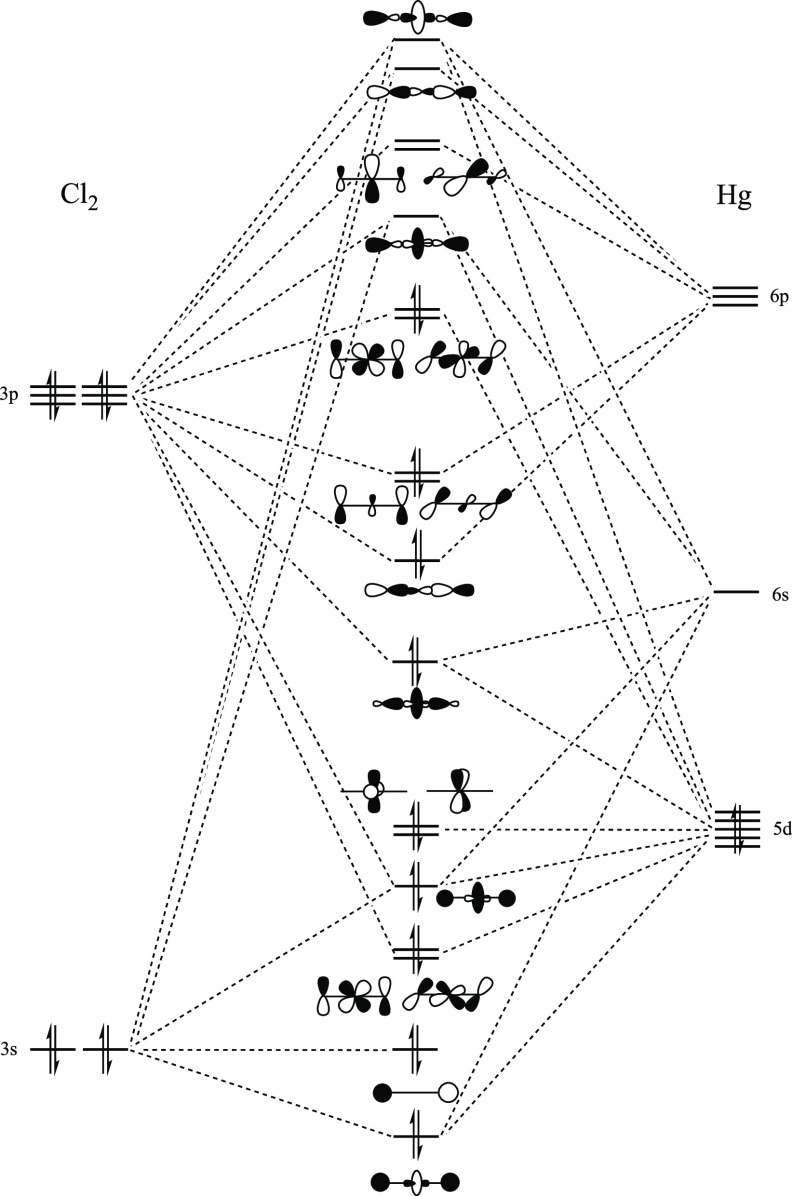
Qualitative molecular orbitals diagram of the HgCl_2_ molecule.

In Figure 5, we show a qualitative molecular orbital diagram for a simple dicoordinated Hg(II) complex, namely, HgCl_2_. It is known that the 6s orbital in mercury, as in other heavy metals, is contracted due to relativistic effects, which makes its hybridization with empty 6p orbitals difficult because of the large energy difference. In fact, the predominance of linear dicoordination in Hg(II) complexes can be explained by the absence of s–p hybridization. A consequence of this is the presence of two degenerate antibonding π* orbitals (LUMO + 1 and LUMO + 2 in Figure 5) that, a priori, can act as charge transfer acceptors from an LB.

**Figure 6:**
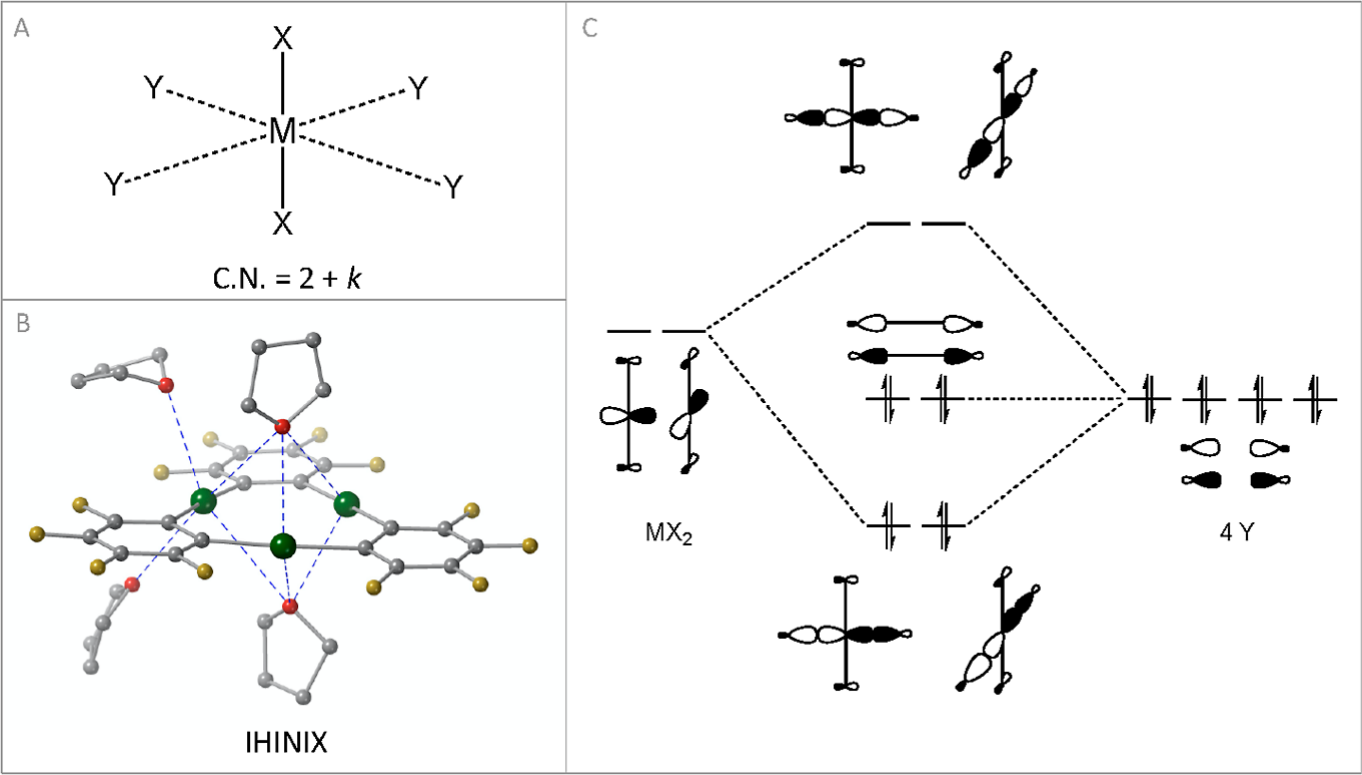
(a) Characteristic and effective coordination spheres in pseudo-octahedral HgX_2_Y_4_ complexes, (b) example of pseudo-octahedral coordination in Hg_3_ anticrowns found in the crystal structure of IHINIX,^40^ and (c) molecular orbitals diagram for the formation of the effective coordination sphere in MX_2_ cores.

The presence of a set of two empty p orbitals at each mercury cation should, in fact, allow its interaction with up to four additional LBs, yielding a linear geometry (coordination number = 2) at the typical coordination bond distances and *k* (*k* = 1–4) extra ligands at longer distances that are however shorter than the vdW radii sum (Figure 6a). This explains the persistence among Hg(II) complexes of geometries such as T-shaped (*k* = 1) or octahedral (*k* = 4). The idea of a double coordination sphere in Hg(II) complexes, comprising a characteristic and effective coordination (2 + *k*), is not new since it was proposed by Grdenić in 1965.^37^ Early theoretical work by Kaupp and von Schnering further investigated this particular behavior in HgX_2_ dimers (X = F, Cl, Br, I, and H).^38,39^ If we look at Hg_3_ anticrowns, there are several examples in the CSD with a linear Hg(II) center in (*o*-C_6_F_4_Hg)_3_ interacting with up to four additional LBs. For instance, in the crystal structure of IHINIX,^40^ one of the dicoordinated mercury centers interacts with four tetrahydrofuran molecules (Figure 6b), resulting in a pseudo-octahedral coordination geometry with the four additional LBs forming a slightly distorted square-planar structure (continuous shape measure *S*_(SP-4)_ = 2.53). This clearly indicates that the set of two empty p orbitals of Hg dictates the final geometry of these 2 + *k* coordination complexes. In fact, the formation of a reverse sandwich-like structure, with two LBs (each binding the three mercury atoms) above and below the anticrown, is a recurrent pattern in the CSD.

The interaction of Hg_3_ anticrowns and electron-rich species has been extensively investigated by the Gabbaï group. They rationalized such interactions in terms of both electrostatics and orbital charge transfer,^41^ proposing for the first time the participation of mercury 6p orbitals as electron density acceptors.^14^ Moreover, a nice example of how orbital interactions can determine the final geometry of an adduct was reported in 2003 by Tsunoda and Gabbaï.^23^ They presented crystallographic evidence of the interaction of two (*o*-C_6_F_4_Hg)_3_ molecules with dimethyl sulfide involving a Hg_3_ centroid–S–Hg_3_ centroid angle of 135.6°, which is the result of the interaction of the sulfur lone pairs with the two sets of three mercury p orbitals above and below the dimethyl sulfide molecule.

In previous reports, the interaction of linear group 12 complexes with extra ligands has been theoretically studied within the π-hole bonding framework.^42,43^ On the other hand, in the molecular orbital theory, the effective coordination sphere can be rationalized in terms of the Rundle–Pimentel model, in which two 3c–4e bonds are formed (Figure 6c). The interaction of the two empty p orbitals of the MX_2_ framework with four lone pairs (4Y) from the LBs forms two bonding, two non-bonding, and two antibonding molecular orbitals that leads to the MX_2_Y_4_ complex, yielding a net energy stabilization of the system, which allows understanding the generalized presence of Hg(II) complexes with a 2 + *k* coordination in the solid state.

### Natural Resonance Theory and Natural Bond Orbital Analyses

As previously pointed out by Coulson^44^ and then by Weinhold,^45^ the electron density distribution associated with the 3c–4e Rundle–Pimentel model for linear systems can be equally described by the resonating ω-bonding model within the natural bond orbital (NBO) formalism.^46^ Accordingly, we have applied the natural resonance theory (NRT)^47^ to the simple HgCl_2_ complex studied above. NRT calculations have disclosed that the system is better described as a resonance 3c/4e triad Cl: Hg–C ⇔ C–Hg: Cl arising from hyperconjugation interactions, with resonance weightings for each of the two resonance structures of 50% and a natural atomic valence for Hg of 1.0. This bonding picture is in good agreement with previous NRT analyses in related transition metal systems.^48,49^ Further exploratory NRT calculations on the F_5_C_6_–Hg–C_6_F_5_ complex, closer to the Hg_3_ anticrown, disclose a more complex bonding scenario with multiple contributing resonant structures, with those that involve C: Hg–C ⇔ C–Hg: C resonance accounting for up to 60% of the resonant weighting.

**Figure 7:**
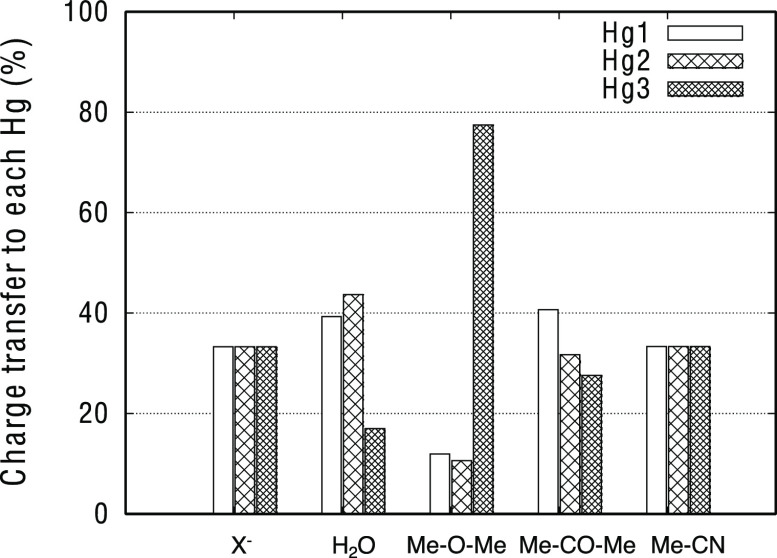
Amount of charge transfer (in percentage) to each of the three mercury centers for the different LBs.

Keeping this in mind, we have next carried out a comprehensive NBO analysis of all the studied adducts. The results are presented in Table 3. Remarkably, in all cases, the acceptor orbitals are σ_Hg–C_^*^ orbitals since the NBO scheme considers the C–Hg–C moieties as 3c/4e hyperbonds, as in the Hg–Cl case described above. The largest second-order perturbation energies (*E*^(2)^) are found for hydride, which is the LB with the highest degree of penetration among all studied adducts (*p*_H–Hg_ = 75.4%), in good agreement with the very large charge transfer term calculated by means of the EDA above. For halides, the charge transferred is the same toward each of the three Hg centers and it does not vary significantly as we descend the group, accounting for a total *E*^(2)^ of 20.1 kcal/mol for iodide. For LBs with oxygen as the donor atom (i.e., water, dimethyl ether, and acetone), more differences are found between the three Hg centers. In the case of acetone, the charge transfer is similar toward the three metal centers. For dimethyl ether, one metal center accepts 77.5% of the total amount of charge transfer, while the other 22.5% is equally distributed between the other two Hg centers. Finally, in the case of water, there are two Hg centers that accumulate more than 80% of the total charge transfer (39.3 and 43.7%, respectively). When the LB is acetonitrile, the donor orbital is an sp hybrid that delocalizes electron density into the σ antibonding orbital of each Hg cation (33.3% of the total CT to each Hg), and the total *E*^(2)^ is 1 order of magnitude smaller than those calculated for halides. A comparative graphical summary of these processes can be seen in Figure 7.

**Table 3:** Donor–Acceptor Orbital Interactions and Associated Second-Order Perturbation Energies (*E*^(2)^) Obtained by Means of NBO analysis for the Adducts Formed by (*o*-C_6_F_4_Hg)_3_ and Different LBs

LB	donor	acceptor	*E*^(2)^ (kcal/mol)
H^–^	*n*_H_ (99% s)	σ_Hg1–C_^*^	25.23
		σ_Hg2–C_^*^	24.27
		σ_Hg3–C_^*^	24.65
F^–^	*n*_F_^a^	σ_Hg2–C_^*^	6.63
		σ_Hg3–C_^*^	6.57
		σ_Hg3–C_^*^	6.57
Cl^–^	*n*_Cl_	σ_Hg1–C_^*^	6.49
		σ_Hg2–C_^*^	6.55
		σ_Hg3–C_^*^	6.54
Br^–^	*n*_Br_	σ_Hg1–C_^*^	6.65
		σ_Hg2–C_^*^	6.65
		σ_Hg3–C_^*^	6.65
I^–^	*n*_I_	σ_Hg1–C_^*^	6.80
		σ_Hg2–C_^*^	6.78
		σ_Hg3–C_^*^	6.79
H_2_O	*n*_O_ (52% s; 48% p)	σ_Hg1–C_^*^	0.48
		σ_Hg2–C_^*^	0.55
		σ_Hg3–C_^*^	0.35
	*n*_O_ (99% p)	σ_Hg1–C_^*^	0.33
		σ_Hg2–C_^*^	0.35
Me–O–Me	*n*_O_ (45% s; 55% p)	σ_Hg1–C_^*^	0.10
		σ_Hg2–C_^*^	0.10
		σ_Hg3–C_^*^	1.01
	*n*_O_ (99% p)	σ_Hg1–C_^*^	0.08
		σ_Hg2–C_^*^	0.06
		σ_Hg3–C_^*^	0.16
Me–CO–Me	*n*_O_ (58% s; 42% p)	σ_Hg1–C_^*^	0.71
		σ_Hg2–C_^*^	0.41
		σ_Hg3–C_^*^	0.36
	*n*_O_ (99% p)	σ_Hg1–C_^*^	0.38
		σ_Hg2–C_^*^	0.44
		σ_Hg3–C_^*^	0.38
Me–CN	*n*_N_ (53% s; 47% p)	σ_Hg1–C_^*^	0.74
		σ_Hg2–C_^*^	0.76
		σ_Hg3–C_^*^	0.75

a The values of *E*^(2)^ correspond to the sum of the electron transfer from each of the four lone pairs in each halide anion.

The different behaviors of the studied LBs with respect to the charge transfer distribution between the three metal centers can be explained in terms of the shape and size of the involved orbitals. We have mentioned before that the acceptor orbitals are empty σ antibonding Hg–C orbitals. On the other hand, the electron density donor orbitals involved vary in composition and number. In the case of O-donor LBs, there are two lone pairs involved as donors, one is an sp hybrid, while the other one is a pure p orbital. We think that, in these cases, the presence of these p lone pairs determines the geometry of the adducts. For an optimum overlap of these with the acceptor orbitals, the LB must be closer to two mercury cations. This explains the larger out-of-center displacement found, both experimentally (Figure 4) and computationally (Table 1), for R_2_O donors.

For halides, the four lone pair orbitals present some interesting features. There are two p lone pairs that remain non-hybridized (99.9% p), while the remaining p and the s lone pairs mix to different degrees to form two hybrids, s^*x*^p^*y*^ and s^*y*^p^*x*^. The degree of sp hybridization is maximum for F (*x* = 0.85, *y* = 0.15) and minimum for I (*x* = 0.95, *y* = 0.05). If we look back at the EDA, while the charge transfer is practically the same for all halides and the changes in the Frozen term are small, the polarization term, associated with the intrafragment reorganization of the molecular orbitals, significantly decreases when descending the group and shows a nice linear correlation with the sp hybridization degree seen in the NBO orbitals (*R*^2^ = 0.99; see Figure S2 in the Supporting Information). Thus, we can conclude that the internal molecular orbital relaxation, that is, rehybridization, of the halide anions upon interaction is responsible for (1) the large interaction energies computed for halides and (2) the significant decrease in the interaction strength when descending the halogen group.

## Conclusions

The performance of Hg_3_ anticrowns, in particular, (*o*-C_6_F_4_Hg)_3_, as anion capturers has been evaluated by means of structural and computational analyses. CSD searches have disclosed that these systems are able to bind a large range of electron-rich species, including anionic and neutral LBs. The MEP of the anticrown has clearly shown the Lewis acidic character of the central part of the molecule. We have calculated remarkably large interaction energies with monoatomic anions, showing less affinity for neutral species often used as solvents, for instance, water or acetonitrile. Although the electrostatic analysis predicts the interaction of the LB with the center of the anticrown, we have observed an unexpected out-of-center displacement of the binding site for some LBs, which we have been able to explain by means of an analysis of the orbitals involved in the interaction. Furthermore, a generalization of the findings in terms of the molecular orbital theory and NBO formalism has helped us understand the effective coordination sphere in linear mercury complexes. These results will be useful for the development of improved anion receptors based on polydentate macrocyclic systems and to further understand the peculiar coordination chemistry of d^10^ closed-shell heavy metals such as Hg(II) and Au(I).

## Computational Methods

Structural searches were performed in the Cambridge Structural Database (CSD)^50^ version 5.41 (November 2019) + 3 updates, allowing only crystal structures with 3D coordinates determined, with no errors and an R factor smaller than 0.1. All electronic structural calculations were carried out using Gaussian16.^51^ Based on previous benchmark studies on mercury complexes,^52^ we employed the hybrid B3LYP functional with Grimme’s empirical dispersion and Becke–Johnson dumping (B3LYP-D3BJ) and triple quality def2-TZVPD basis sets for all atoms, including the corresponding relativistic pseudopotentials for heavy atoms. All systems in Table 1 were fully optimized and confirmed to be real minima of the corresponding PESs by means of vibrational analysis. Interaction energies were calculated via the supermolecule approach [Δ*E*_int_ (AB) = *E*_AB_ – (*E*_A_ + *E*_B_)] at the DLPNO-CCSD(T) level on the DFT-optimized systems without the correction of the basis set superposition error.^53^ MEP maps were built using GaussView^54^ on the 0.001 au isosurface. The interaction energies were decomposed into chemically meaningful terms (Δ*E*_INT_ = Δ*E*_FRZ_ + Δ*E*_POL_ + Δ*E*_CT_) by means of the second-generation ALMO-EDA procedure as implemented in QChem5^55^ at the B3LYP-D3BJ level. The presence of pseudopotentials to describe heavy atoms forced us to apply the classical decomposition of the frozen energy (Δ*E*_FRZ_ = Δ*E*_Pauli_^MOD^ + Δ*E*_Elect_^CLS^ + Δ*E*_Disp_^CLS^). NRT and NBO analyses were performed using the NBO7 program^56^ on the optimized geometries. Molecular orbital diagrams are qualitative and based on the Khon–Sham orbitals calculated at the B3LYP level.

## Supporting Information Available

Linear correlations between energies and penetrations and hybridizations (PDF)
Cartesian coordinates of all optimized systems (XYZ)

## Supplementary Material

ic2c00921_si_001.pdf

ic2c00921_si_002.xyz
