# Supplementary material for: Anion Binding Based on Hg3 Anticrowns as Multidentate Lewis Acidic Hosts
Source: Inorg Chem. 2022 Aug 1;61(32):12526–33. doi: 10.1021/acs.inorgchem.2c00921 (PMC9948291; doi:10.1021/acs.inorgchem.2c00921)
Supplement: Supplementary file 1 — ic2c00921_si_001.pdf [file ic2c00921_si_001.pdf]

SUPPORTING INFORMATION

Anion binding based on Hg_3-anticrowns as multidentate Lewis acidic hosts

Oliver Loveday,[a] Jesús Jover[a] and Jorge Echeverría[b]*

a) Secció de Química Inorgànica, Departament de Química Inorgànica i Orgànica and Institut de Química Teòrica i Computacional (IQTC-UB), Universitat de Barcelona, Martí i Franquès 1-11, 08028, Barcelona (Spain).

b) Departamento de Química Inorgánica, Instituto de Síntesis Química y Catálisis Homogénea (ISQCH), CSIC-Universidad de Zaragoza, Pedro Cerbuna 12, 50009 Zaragoza (Spain).

e-mail: jorge.echeverria@unizar.es

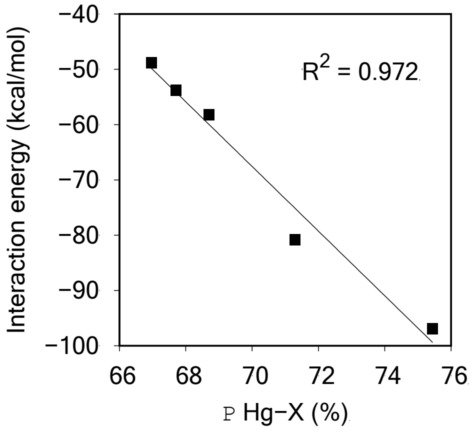

Figure S1. Linear correlation between the interaction energies calculated for anions ($X = H^-$, F^-, Cl^-, Br^- and I^-) and the degree of penetration between Hg and X.

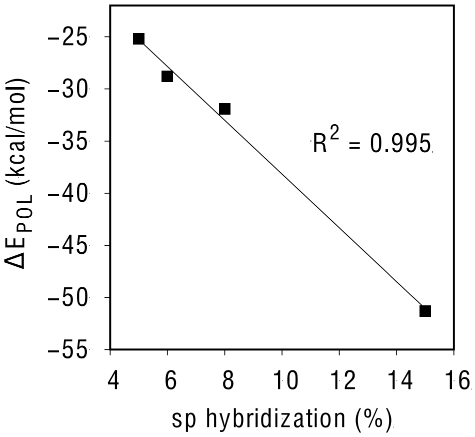

Figure S2. Linear correlation between the polarization term of the EDA and the degree of *sp* hybridization of the two halide lone pairs in the natural localized orbitals.
